# Carbon Market Price Forecasting Using a Bidirectional Temporal Convolution Exogenous-Enhanced Time-Series Model

**DOI:** 10.3390/e28070822

**Published:** 2026-07-19

**Authors:** Xinyu Tang, Mingzhu Tang, Na Li, Shumei Zhang

**Affiliations:** 1 School of Electrical and Information Engineering, Tianjin University, Tianjin 300072, China; tangxinyu@tju.edu.cn (X.T.); shumeizhang@tju.edu.cn (S.Z.); 2 School of Energy and Power Engineering, Changsha University of Science & Technology, Changsha 410114, China; 3 School of Economics and Management, Changsha University of Science & Technology, Changsha 410114, China; nikki_lina@163.com

**Keywords:** carbon market price forecasting, bidirectional temporal convolutional network, TimeXer, cross-attention, multivariate time-series modeling

## Abstract

Carbon market prices are jointly shaped by policy interventions, energy market fluctuations, and macroeconomic dynamics, and thus exhibit pronounced nonlinearity, non-stationarity, localized abrupt changes, and time-varying uncertainty. From an information-theoretic perspective, carbon price forecasting can be viewed as the extraction and fusion of effective information from a complex market system driven by heterogeneous endogenous and exogenous signals. To address the challenges of accurately characterizing local high-frequency fluctuations in carbon price series, effectively modeling the interactions between endogenous and exogenous variables, and mitigating the structural noise introduced by conventional serial forecasting frameworks, this study proposes ConvTimeXer, a hybrid model combining bidirectional temporal convolution and TimeXer for carbon market price forecasting. Specifically, the model first employs front-end bidirectional temporal convolutions to extract local multi-scale fluctuation features from the endogenous carbon price series. It then leverages the global token and cross-attention mechanism in TimeXer to achieve dynamic decoupling and deep interaction between endogenous and exogenous variables. Finally, residual fusion of shallow and deep features is introduced to enhance the preservation of local details. Experimental results based on data from China’s carbon market over the past three years demonstrate that the proposed framework delivers high predictive accuracy and strong robustness, effectively balancing responsiveness to local abrupt changes with global trend modeling. This study not only provides an effective approach for carbon price forecasting in complex and uncertain market environments, but also offers valuable insights into non-stationary time-series forecasting driven by multi-source heterogeneous information.

## 1. Introduction

Accurate carbon market price forecasting provides an important basis for governments and enterprises to formulate effective emission-reduction policies and market regulation mechanisms [1]. As market mechanisms become increasingly complex, carbon price series exhibit pronounced nonlinearity, non-stationarity, abrupt fluctuations, and uncertainty. Traditional time-series forecasting methods, such as ARIMA and Holt–Winters models, are primarily designed for univariate settings, which limits their ability to capture such complex temporal dynamics [2]. Consequently, developing forecasting models that simultaneously achieve high accuracy, robustness, and generalization capability has become a critical issue for both governments and enterprises, as well as a representative problem in the analysis of complex market systems.

From the perspective of information theory and complex systems, carbon price formation can be regarded as an information-processing process driven by heterogeneous endogenous and exogenous signals. Policy interventions, energy-price shocks, macroeconomic conditions, and financial-market fluctuations jointly reshape the information structure of the carbon market, leading to time-varying uncertainty, nonlinear dependencies, and regime-dependent volatility. Therefore, carbon price forecasting is not only a conventional prediction task, but also a problem of extracting effective information from a complex, non-stationary system under uncertainty.

Entropy-based and information-theoretic measures have been widely used to characterize uncertainty, complexity, irregularity, and information transfer in nonlinear time series. Measures such as Shannon entropy, sample entropy, permutation entropy, and transfer entropy provide useful conceptual tools for quantifying unpredictability, detecting hidden temporal structures, and analyzing directional information interactions among coupled variables [3,4,5,6]. For carbon markets, such perspectives are particularly relevant because price dynamics are affected by multiple interacting subsystems, including energy markets, macroeconomic conditions, financial variables, and policy environments.

In recent years, carbon price forecasting has gradually shifted toward hybrid modeling paradigms that combine signal decomposition with deep learning. Ni et al. (2024) improved the predictability of carbon price sub-series through a combined complementary ensemble empirical mode decomposition (CEEMD) and variational mode decomposition (VMD) framework and subsequently introduced neural networks for forecasting, thereby effectively reducing prediction errors [7]. To address local fluctuations in regional markets, Shi et al. (2024), in a study of the Shenzhen carbon market, showed that a convolutional neural network (CNN)–long short-term memory (LSTM) architecture incorporating a decomposition mechanism outperformed single recurrent networks in both accuracy and robustness [8]. Qin et al. (2024) further demonstrated that bidirectional long short-term memory (BiLSTM) enhanced with an attention mechanism improves the identification of complex dynamic patterns [9]. In addition, some studies have employed empirical wavelet transform or improved secondary decomposition algorithms in conjunction with multi-head attention and BiLSTM networks to improve forecasting accuracy by effectively stripping high-frequency noise from non-stationary series [10,11,12,13]. More recently, Wei et al. (2025) proposed a multifractal-aware convolutional attention forecasting framework for carbon market prices and showed that multi-scale convolutional feature extraction can further enhance predictive performance under complex nonlinear fluctuations [14,15,16,17]. Nevertheless, such approaches typically rely on a multi-stage serial pipeline of decomposition, forecasting, and reconstruction. In real-time rolling forecasting, boundary distortions generated by the upstream decomposition module can easily propagate into subsequent networks, leading to severe error accumulation [7,18,19,20,21].

Modeling exogenous factors has become another important direction for improving carbon price forecasting performance. Carbon price evolution is not a closed autoregressive process; rather, it is continuously shaped by external forces such as energy prices, macroeconomic cycles, and market sentiment. Based on this understanding, multivariate collaborative forecasting has gradually become a central research focus. Zhang et al. (2024) integrated multidimensional influencing factors into a hybrid multivariate forecasting framework using LASSO-based feature selection [22]. Huang and Zhang (2024) proposed a conditional generative adversarial network that incorporates multi-source information for carbon price forecasting [23]. At the architectural level, the dual-stream Transformer–attention fusion network proposed by Wu and Du separately modeled inter-variable correlations and temporal dependencies, thereby improving short-term forecasting performance [24]. Furthermore, event-enhanced pre-trained models [25], graph neural network approaches that incorporate spatial dependence [26], and multitask frameworks integrating news text and search behavior data [27] all indicate that the effective organization of exogenous information can not only substantially improve forecasting accuracy, but also enhance model robustness under periods of high market volatility and extreme events, while effectively capturing dynamic interactions among external factors. More recent studies have shown that variables related to technology, finance, and the environment can further improve carbon price forecasting performance [28,29]. From the standpoint of unified feature extraction and deep-learning integration, some studies have shown that effectively selecting and fusing multi-source external factors can enhance the ability of carbon price forecasting models to represent complex nonlinear relationships [30,31]. Others have incorporated multi-frequency exogenous factors into Transformer architectures and demonstrated that the systematic integration of heterogeneous-frequency and multi-factor information contributes to greater stability and accuracy in carbon price trend forecasting [32,33]. However, existing multivariate fusion schemes still largely rely on hidden-layer concatenation or shallow weighting, and lack a unified mechanism capable of dynamically coordinating the relationship between endogenous historical states and exogenous shocks.

Advances in general time-series forecasting have also offered important insights for carbon price modeling. In recent years, the field of general time-series forecasting has developed along a relatively clear trajectory centered on long-sequence modeling, multivariate dependency learning, and distribution-shift adaptation. Representative models such as Informer [34], Autoformer [35], Temporal Fusion Transformer [36], and N-HiTS [37] improve the efficiency of long-sequence and multi-horizon trend modeling through sparse attention, sequence decomposition, covariate-aware fusion, or hierarchical interpolation. However, carbon markets are highly susceptible to policy interventions and extreme events, and therefore exhibit strong non-stationarity and pronounced local high-frequency fluctuations, under which such models are prone to prediction lag. To compensate for insufficient local feature extraction, PatchTST [38] and MICN [39] introduced patching mechanisms and local convolutions to better preserve local semantics. Meanwhile, to address multivariate dependencies, Crossformer [40] and iTransformer [41] attempted to reconstruct attention mechanisms along spatial and variable dimensions.

Nevertheless, existing general-purpose time-series models still suffer from two major limitations when directly applied to carbon price forecasting. First, most models implicitly treat all input variables equally within a closed-system setting, making it difficult to structurally distinguish the endogenous evolution of carbon prices from the driving effects of external market shocks. As a result, structural noise is easily introduced during multi-source information fusion, and the underlying information structure of the carbon market may be obscured. Second, although pure Transformer architectures excel at modeling long-range global dependencies in long sequences, they are less sensitive to sudden, asymmetric, and high-frequency local fluctuations in carbon price series, often resulting in insufficient characterization of local details and uncertainty-driven short-term irregularities. More fundamentally, the key challenge in carbon price forecasting lies in how to jointly model multi-source heterogeneous information while balancing long-range global dependency learning with the representation of intense local fluctuations in a complex and uncertain market system. Existing studies have also shown that Transformers are not always superior in time-series tasks [42], whereas the effectiveness of alternative architectures such as SCINet [2] and temporal convolutional networks [43,44,45,46] further suggests that convolutional structures retain irreplaceable advantages in local interaction modeling and high-frequency feature extraction. Related multivariate attention-based encoder–decoder designs also highlight the value of structured cross-variable interaction under exogenous information [47].

Among recent forecasting architectures, TimeXer is particularly relevant to this study because it explicitly distinguishes endogenous target series from exogenous variables and uses patch-wise self-attention together with variate-wise cross-attention to ingest external information [48]. This separation is useful for carbon-price forecasting because carbon prices are not driven only by their own historical values, but are also affected by energy prices, financial indicators, macroeconomic conditions, and policy-related signals.

To address the above issues, this study proposes ConvTimeXer, a hybrid model integrating a bidirectional temporal convolutional network (BiTCN) with TimeXer [48]. First, front-end bidirectional temporal convolution is employed to capture local multi-scale fluctuation patterns and refine the local information structure of endogenous price dynamics. Then, the TimeXer module is leveraged to achieve deep interaction and dynamic decoupling between endogenous and exogenous variables, thereby selectively aggregating informative external signals under market uncertainty. Finally, shallow-deep feature fusion is utilized to preserve local details while modeling long-range temporal dependencies. Therefore, ConvTimeXer is designed to combine local fluctuation extraction with structured exogenous-variable interaction, so as to improve one-step-ahead carbon price forecasting under non-stationary market conditions.

(1) Information-aware BiTCN-based local refinement

ConvTimeXer introduces a BiTCN-based local refinement module to model abrupt, asymmetric, and high-frequency fluctuations in endogenous carbon price series. Through forward and backward dilated causal convolutions, the module captures multi-scale local dynamics, alleviates the limitations of unidirectional temporal modeling, and performs preliminary smoothing and structural reorganization before deep feature learning. This design enhances the representation of local information structures under non-stationary market uncertainty.

(2) Dynamic endogenous-exogenous information interaction

To model the joint effects of endogenous evolution and exogenous shocks, the framework incorporates a TimeXer-based interaction mechanism that dynamically decouples endogenous and exogenous information. By combining patch-based representation, a learnable global token, and cross-attention, the model selectively aggregates the external signals most relevant to the current carbon price trajectory, thereby reducing redundant information and structural noise caused by direct feature concatenation.

(3) Complexity-oriented shallow-deep residual fusion

To preserve local fluctuation details that may be attenuated during deep feature extraction, ConvTimeXer introduces a shallow–deep residual fusion strategy at the output stage. Specifically, the local features refined by BiTCN and aggregated into patch-level representations are residually fused with the deep global representations generated by TimeXer. This design retains short-term, high-frequency information while incorporating long-range contextual dependencies, thereby enabling the model to better balance sensitivity to abrupt local fluctuations with stability in global trend modeling under non-stationary market conditions.

The specific aims of this study are therefore threefold: to improve the representation of abrupt local carbon-price fluctuations, to model exogenous market information through an explicit cross-attention pathway, and to evaluate whether the resulting model can achieve stable one-step-ahead forecasting performance under leakage-free time-series validation. The results show that these aims are achieved through the complementary effects of BiTCN-based local refinement, TimeXer-based endogenous-exogenous interaction, and residual shallow-deep feature fusion.

The remainder of this paper is structured as follows: Section 2 presents the methodology of the proposed ConvTimeXer framework, including the problem formulation, data preprocessing, model architecture, loss function, and leakage-free evaluation strategy. Section 3 describes the experimental design, including the evaluation metrics and parameter settings. Section 4 reports the comparative and ablation results and discusses the forecasting performance of the proposed model. Section 5 concludes the paper and outlines its limitations and future research directions.

## 2. Methodology

### 2.1. Problem Statement

Carbon price forecasting can be formulated as a multivariate time-series prediction task in which the future carbon price is determined by both endogenous historical dynamics and exogenous market information [1]. Owing to policy interventions, energy-price fluctuations, and macroeconomic disturbances, carbon price series typically exhibit strong nonlinearity, non-stationarity, and localized abrupt changes. These characteristics require the forecasting model to preserve sensitivity to short-term fluctuations while capturing long-range temporal dependencies and cross-variable interactions under strict temporal-causality constraints. At the same time, carbon price evolution is not a closed autoregressive process, and multidimensional exogenous factors have been shown to significantly improve forecasting performance [14,24].

Accordingly, the objective of this study is to construct a unified end-to-end framework that can effectively model local endogenous fluctuations, selectively incorporate exogenous signals, and maintain stable generalization in rolling forecasting scenarios.

### 2.2. Data Preprocessing and Feature Extraction

To address the multi-scale characteristics of carbon price fluctuations, this study constructs a multi-dimensional feature set. First, time covariates such as monthly and quarterly intervals are extracted from the original transaction timestamps and mapped into continuous periodic functional features to explicitly capture the inherent seasonal rhythms of the carbon market. Second, an external feature library is constructed using coal prices, crude oil prices, and macroeconomic indices. Finally, the feature space is rigidly decoupled into endogenous and exogenous components based on physical significance. As shown in Table 1, the exogenous variables consist of all covariates except date and price and are denoted as *x_exo_*, where *x_exo_* represents the vector of external market and macroeconomic covariates. The endogenous variable consists solely of the carbon price series (corresponding to the data column price) and is denoted as *x_endo_*, where *x_endo_* represents the target-channel historical price sequence.

To ensure the convergence stability of model training and the reliability of prediction results, this study performed data preprocessing and feature extraction on the original multidimensional data. The specific steps are as follows:

(1) Z-score standardization. Since the endogenous and exogenous variables use different units of measurement and their numerical scales differ significantly, Z-score standardization is performed to eliminate scale effects and enhance the comparability of the variables:

(1) $${\tilde{p}}_{t}=\frac{p_{t}-\mu^{\left(p\right)}}{\sigma^{\left(p\right)}}$$

(2) $${\tilde{z}}_{k,t}=\frac{z_{k,t}-\mu^{\left(k\right)}}{\sigma^{\left(k\right)}},\;k=1,\ldots \ldots ,14$$

(2) Sliding window and supervised samples. Let the historical window length be T = 30 and the forecasting horizon be H = 1. Indexing samples by the starting position i, the input of the i-th sample is defined as $X_{i}^{endo}=[{\tilde{p}}_{i},\ldots \ldots ,{\tilde{p}}_{i+T-1}{]}^{⊤}\in R^{T\times 1}$, $X_{i}^{exo}=[{\tilde{z}}_{i}^{⊤},\ldots \ldots ,{\tilde{z}}_{i+T-1}^{⊤}{]}^{⊤}\in R^{T\times 14}$, and the supervisory signal is the scalar *yᵢ*, corresponding to the carbon price at the next time step after the window following target-channel standardization. In the forward pass of the network, the two inputs are denoted as *x_endo_* and *x_exo_*.

(3) Reversible instance normalization (RevIN) within each window [49]. Before being fed into the BiTCN, the endogenous sequence in each window is normalized along the temporal dimension, with the following statistics computed:

(3) $$\mu_{i}=\frac{1}{T}{\sum_{\tau =0}^{T-1}\hat{p}}_{i+\tau }$$

(4) $$\sigma_{i}^{2}=\frac{1}{T}\sum_{\tau =0}^{T-1}({\hat{p}}_{i+\tau }-\mu_{i}{)}^{2}$$

Here, $\hat{p}$ represents the Z-score-normalized carbon price, and thus

(5) $${\overset{ˇ}{p}}_{i+\tau }=\frac{{\hat{p}}_{i+\tau }-\mu_{i}}{\sigma_{i}+\varepsilon }$$

After the prediction is generated, the output is recovered via the inverse transformation:

(6) $$pred=\hat{o}\cdot \sigma_{i}+\mu_{i}$$

The exogenous channels are standardized only using Z-score normalization, without applying RevIN.

Missing-value handling and chronological dataset partitioning are described in Section 3.1.

### 2.3. Construction of the ConvTimeXer Forecasting Framework

For clarity, the framework should be interpreted as an end-to-end forecasting architecture rather than a serial decomposition-reconstruction pipeline. The endogenous branch carries the historical carbon price sequence, whereas the exogenous branch carries market and macroeconomic covariates. These two streams are kept structurally separate until the TimeXer cross-attention stage, which reduces the risk of introducing structural noise through direct feature concatenation.

**Figure 1:**
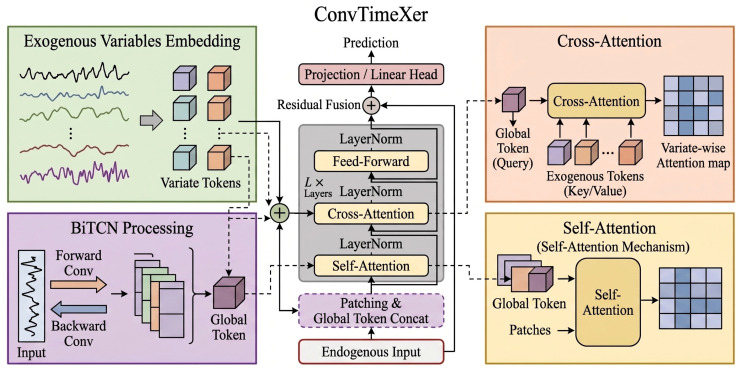
Workflow of the ConvTimeXer forecasting framework.

To address the challenges posed by the complex non-stationary fluctuations of carbon price series and the difficulty of jointly modeling endogenous and exogenous variables, this study develops a hybrid forecasting framework, termed ConvTimeXer, which combines a BiTCN-based temporal representation module with a TimeXer-based forecasting module. Departing from conventional multi-stage serial decomposition pipelines, the proposed framework adopts a three-stage collaborative modeling strategy consisting of local feature refinement, global cross-variable interaction, and shallow-to-deep residual fusion. The overall workflow and architectural design of ConvTimeXer are illustrated in Figure 1 and described as follows.

In the local feature refinement stage, the model introduces BiTCN to address the frequent local abrupt changes, intense short-term volatility, and pronounced asymmetry in endogenous carbon price series. By employing forward and backward dilated causal convolutions, this module overcomes the limitations of unidirectional temporal processing and adaptively extracts multi-scale high-frequency fluctuations and local abrupt-change patterns from the carbon price series before the features are passed to deeper network layers. In doing so, it achieves preliminary smoothing and structural reorganization of short-term non-stationary fluctuations.

In the global cross-variable interaction stage, the model leverages the TimeXer architecture to achieve dynamic decoupling of endogenous and exogenous information. The refined temporal features output by BiTCN are segmented into sequential patches, and a learnable global token is appended to the end of the patch sequence. The resulting token sequence is then fed into a self-attention layer to capture long-range temporal dependencies. Meanwhile, the exogenous variables are independently mapped into variable tokens. Subsequently, the global token serves as an information interaction hub and, through the cross-attention mechanism, actively selects and aggregates those external environmental signals from the exogenous tokens that are most explanatory of the current carbon price trajectory, thereby effectively avoiding the structural noise introduced by direct concatenation.

As shown in Figure 1, the solid lines represent the main sequential processing flow of ConvTimeXer. Specifically, the endogenous carbon-price sequence is processed by the BiTCN module, converted into patch-level representations, passed through the TimeXer-based attention module, and finally delivered to the residual fusion and forecasting output layer. The dashed lines denote auxiliary information paths rather than independent forecasting branches. They are used to emphasize the exogenous-information interaction pathway through which external variables contribute to the global-token-based cross-attention mechanism, as well as the shallow-to-deep residual/skip connection through which local BiTCN features are preserved and fused with deep TimeXer representations.

In the shallow-to-deep residual fusion stage, skip connections are introduced at the output end of the model to prevent the loss of original local fluctuation details after multiple layers of Transformer-based abstraction. Specifically, the shallow local features extracted by BiTCN are combined residually with the deep global features produced by TimeXer, and the final forecasting results are generated through a fully connected layer. This closed-loop design enables ConvTimeXer to achieve an effective balance between responsiveness to local abrupt changes and global trend modeling within a unified framework.

### 2.4. Core Network Architecture of ConvTimeXer

#### 2.4.1. Bidirectional Temporal Convolutional Feature Extraction

**Figure 2:**
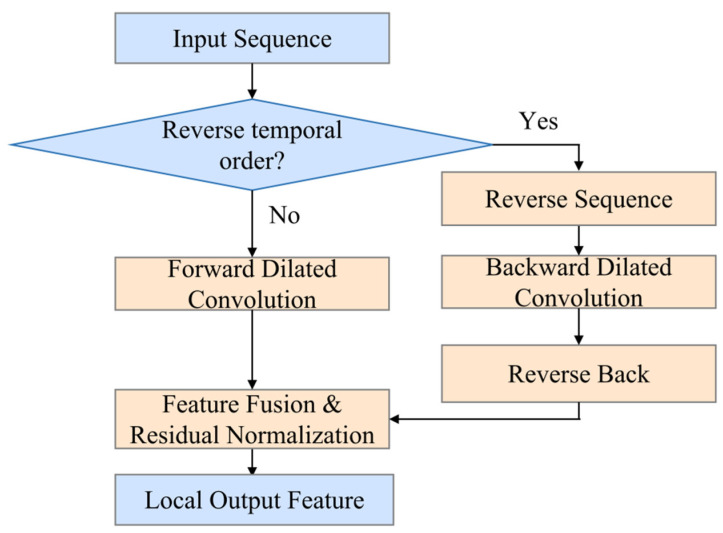
Workflow of bidirectional temporal convolutional feature extraction.

To fully capture the local dependencies and multi-scale evolutionary patterns of the endogenous sequence, the model first employs a temporal convolutional network (TCN) as the basic feature extractor, as illustrated in Figure 2. Compared with sequence models that place greater emphasis on global interactions, TCN is better suited as a front-end local dynamic encoding module for refining the local structural information embedded in short-term carbon price fluctuations [43,44,45,46]. A standard TCN relies on dilated causal convolutions to expand the receptive field. For a one-dimensional sequence input x and a convolution kernel f, the dilated convolution operation is defined as follows:

(7) $$F\left(x_{t}\right)=\sum_{i=0}^{k-1}f\left(i\right)\cdot x_{t-d\cdot i}$$

where d denotes the dilation rate and k denotes the kernel size.

To overcome the limitation of unidirectional causal convolution, which can exploit only historical information, the proposed model introduces a bidirectional mechanism. By reversing the input sequence along the temporal axis, the model computes forward and backward convolutional features simultaneously and then fuses them. In addition, to ensure gradient stability in the deep network, each dilated convolutional layer is equipped with residual connections and layer normalization, formulated as follows:

(8) $$y=LayerNorm\left(x+Dropout\left(F\left(x\right)\right)\right)$$

#### 2.4.2. Sequence Patching and Global Embedding

**Figure 3:**
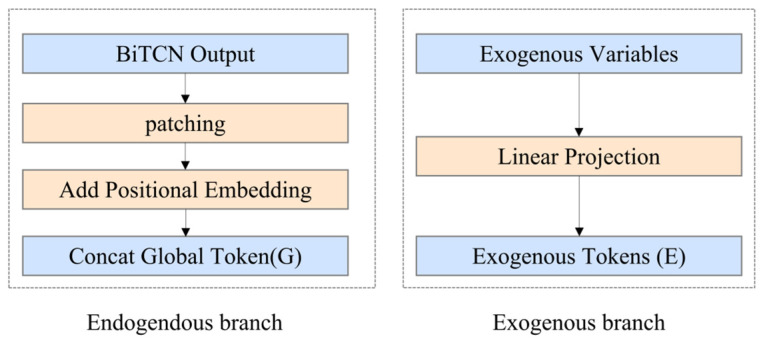
Workflow of sequence patching and global embedding.

To reduce the computational complexity of the subsequent Transformer architecture while extracting local semantics, the model partitions the high-dimensional time-series features generated by the TCN into multiple non-overlapping patches along the temporal dimension, as illustrated in Figure 3. This design is conceptually similar to the patch-based representation adopted in PatchTST to enhance local semantic modeling; however, the present study places greater emphasis on its role in feature compression and structural alignment prior to heterogeneous exogenous modeling. For the endogenous sequence, the dimension-aligned features are projected into a patch sequence and augmented with positional encoding.

Meanwhile, as shown in Figure 3, a learnable global token *G* is appended to the end of the endogenous patch sequence to serve as an information hub in the subsequent cross-attention mechanism between endogenous and exogenous variables. In parallel, the exogenous feature vector at each time step is linearly projected into an exogenous embedding sequence E, thereby preparing it for the subsequent cross-attention operation.

#### 2.4.3. TimeXer Attention Mechanism

**Figure 4:**
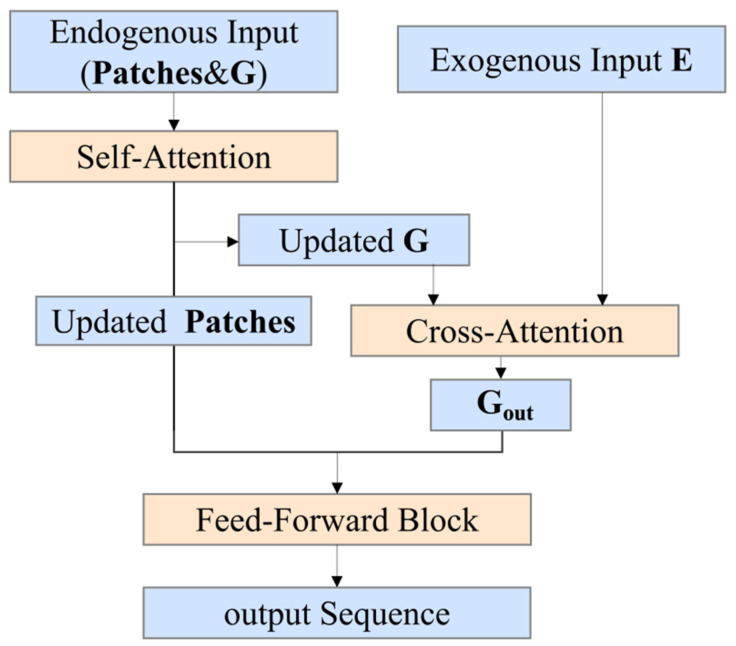
Workflow of the TimeXer attention mechanism.

This module consists of multiple stacked Transformer layers, each comprising two key stages: self-attention and cross-attention, as illustrated in Figure 4. The core computation of the attention mechanism follows the scaled dot-product attention formula, where *Q*, *K*, and *V* denote the query, key, and value matrices, respectively, and *d_k_* denotes the key dimension used for scaling:

(9) $$\mathrm{Attention}\left(Q,K,V\right)=Softmax\left(\frac{QK^{T}}{\sqrt{d_{k}}}\right)V$$

In Figure 4, the bold letter G denotes the learnable global token, and the bold letter E denotes the exogenous token sequence.

First, the endogenous patches and the global token *G* jointly participate in the self-attention computation to capture long-range temporal dependencies. The updated global token *G* is then separated from the resulting representation. To incorporate information from the external market environment, the model uses *G* as the query, while the exogenous token sequence *E* serves as the key and value, yielding the following cross-attention operation:

(10) $$G_{out}=LayerNorm\left(G^{\prime }+CrossAttention\left(G^{\prime },E_{exo},E_{exo}\right)\right)$$

As shown in Figure 4, through this mechanism, the global token can adaptively query and aggregate the most informative external signals from the exogenous variables for the current forecasting task. The updated global token is then appended again to the end of the updated endogenous patch sequence, and the resulting token sequence is processed by the Feed-Forward Block. In Figure 4, this simplified block denotes the FFN together with its residual connection and layer-normalization operation.

#### 2.4.4. Deep Feature Fusion

To prevent the loss of local temporal fluctuation details, the model introduces a skip-connection strategy at the output stage. Specifically, the shallow adaptive features extracted by the early-stage BiTCN and aggregated into non-overlapping patches by averaging within each patch are residually added to the deep global patch features generated by the Transformer, as follows:

(11) $$H_{fusion}=LayerNorm\left(H_{local}+H_{global}\right)$$

where *F_fused* denotes the deep feature representation after residual fusion; *F_shallow* denotes the shallow local adaptive features extracted by the early-stage BiTCN and subsequently aggregated into patch-level representations; *F_deep* denotes the deep global patch features produced by the TimeXer module; and *LN* denotes the layer normalization operation.

Finally, the fused deep features are flattened and fed into a linear fully connected layer to generate the one-step-ahead forecast.

### 2.5. Loss Function and Robust Training Strategy

Given that carbon market price series are often accompanied by severe non-stationary fluctuations and outliers, this study adopts a dedicated loss function and a robust training strategy. Traditional mean squared error is highly sensitive to outliers and may easily lead to gradient explosion or overfitting under extreme market conditions, whereas mean absolute error is non-differentiable in the vicinity of zero. To enhance the model’s robustness to extreme fluctuations, the Huber loss is employed as the objective function for end-to-end optimization, and is defined as follows:

(12) $$L_{\delta }\left(y,\hat{y}\right)=\left\{\begin{array}{ll} \frac{1}{2}(y-\hat{y}{)}^{2}, & for\left|y-\hat{y}\right|\leq \delta \\ \delta \left|y-\hat{y}\right|-\frac{1}{2}\delta^{2}, & otherwise \end{array}\right.$$

where *y* denotes the true carbon price, *ŷ* denotes the predicted carbon price, and *δ* is the smoothing parameter. When the prediction error is small, the loss function takes a quadratic form, thereby accelerating model convergence. When extreme outliers lead to large prediction errors, the loss function automatically transitions to a linear form, effectively suppressing the excessive influence of anomalous samples on gradient updates. In addition, global gradient clipping and an early stopping strategy are employed during training to further prevent overfitting and improve the stability of the optimization process.

### 2.6. Hyperparameter Configuration and Leakage-Free Evaluation Mechanism

Because the available carbon-market sample is limited relative to the flexibility of deep neural forecasting models, overfitting is a central methodological risk. This study mitigates that risk through a combination of chronological validation, early stopping, dropout, weight decay, Huber loss, gradient clipping, and parameter selection based on forward-chaining fold performance. Although these settings cannot fully eliminate the limitations caused by the sample size, they help make the evaluation less dependent on short-term noise and reduce the risk of look-ahead leakage.

To reduce the subjectivity associated with manual hyperparameter tuning, this study conducts a systematic comparison based on the results of time-series cross-validation and determines the optimal hyperparameter combination under the strict preservation of temporal causality. Conventional random data partitioning disrupts the temporal dependence inherent in time-series data, often leading to overly optimistic evaluation results that may fail to generalize in real market applications. To establish a rigorous and realistic evaluation setting, random splitting is discarded during model training, hyperparameter optimization, and final performance assessment, and a forward-chaining time-series cross-validation scheme is introduced instead. This strategy is consistent with recent forecasting studies that emphasize realistic temporal evaluation settings [1,50]. By progressively advancing the training and testing windows over time, it ensures that, in every validation fold, the timestamps in the training set are strictly earlier than those in the test set, thereby eliminating the risk of look-ahead leakage from future data at the algorithmic level and closely approximating a real-world dynamic market environment.

Meanwhile, to further reduce the risk of future information leakage in time-series forecasting, the same forward-chaining time-series cross-validation mechanism is adopted during both hyperparameter optimization and model evaluation. Unlike conventional random partitioning, this mechanism guarantees that, in each training–testing split, the timestamps of the training set always strictly precede those of the test set. The model hyperparameters are primarily determined through time-series cross-validation combined with manual tuning, and the forward rolling test results are then used to comprehensively evaluate the model’s generalization capability.

## 3. Experiments

### 3.1. Dataset Description

The carbon-market price data used in this study were collected from the official website of the China Coal Transportation and Distribution Association (CCTD), and the exogenous variables were obtained from corresponding public market and macro-financial data sources. After data alignment and preprocessing, the integrated empirical dataset consists of 1112 chronologically ordered observations spanning from July 2021 to February 2026. The dataset contains the target carbon price series and a group of representative exogenous variables associated with exchange rates, energy markets, power generation, and macro-financial conditions, thus constituting a multivariate forecasting dataset for China’s carbon market. By jointly incorporating endogenous and exogenous information, the dataset provides an appropriate basis for evaluating the effectiveness of the proposed framework in modeling complex carbon price dynamics.

Exogenous covariates with a missing rate exceeding 30% were removed, whereas those below this threshold were imputed using forward-fill and backward-fill methods. The chronological train/evaluation split was performed using forward-chaining time-series cross-validation, ensuring that, in each fold, the timestamps of the training set strictly preceded those of the evaluation set.

The subsequent comparative evaluation is conducted on three chronological evaluation subsets obtained under the same preprocessing procedure and forward-chaining time-series cross-validation scheme described above. In each subset, earlier observations are used for model fitting, whereas later observations are reserved for out-of-sample evaluation.

Considering the strict temporal dependence of carbon price forecasting, all observations are arranged in chronological order throughout the experimental process. To avoid look-ahead bias and better simulate practical forecasting scenarios, model training and out-of-sample evaluation are implemented under a forward-chaining time-series evaluation scheme, where earlier observations are always used to predict later ones. This setting enables a more realistic and rigorous evaluation of model performance under non-stationary market conditions.

### 3.2. Evaluation Metrics

To objectively assess the performance of the proposed hybrid model in forecasting the target variable, this study adopts mean absolute error (MAE), root mean square error (RMSE), and the coefficient of determination (R^2^) as the primary evaluation metrics. To ensure the validity of the evaluation results, all metrics are computed after inverse transformation to the original price scale.

Mean absolute error (MAE) measures the average absolute deviation between the predicted prices and the true prices. This metric assigns equal weight to all prediction errors and thus provides an intuitive indication of the overall level of prediction deviation on the test set. It is defined as follows:

(13) $$MAE=\frac{1}{n}\sum_{i=1}^{n}\left|y_{i}-{\hat{y}}_{i}\right|$$

Root mean square error (RMSE) is defined as the square root of the mean of the squared prediction errors. Because the residuals are squared during computation, RMSE assigns a larger penalty to larger prediction deviations, and is therefore more sensitive in capturing and reflecting the model’s robustness when dealing with extreme price fluctuations. It is defined as follows:

(14) $$RMSE=\sqrt{\frac{1}{n}\sum_{i=1}^{n}(y_{i}-{\hat{y}}_{i}{)}^{2}}$$

The coefficient of determination (R^2^) is primarily used to measure the extent to which the model explains the variance of fluctuations in the target variable. As a relative evaluation metric, it reflects the goodness of fit of the model to unseen time-series data, with the ideal value being 1. It is defined as follows:

(15) $$R^{2}=1-\frac{\sum_{i=1}^{n}(y_{i}-{\hat{y}}_{i}{)}^{2}}{\sum_{i=1}^{n}(y_{i}-\bar{y}{)}^{2}}$$

In the above equations, *n* denotes the total number of time-series samples in the validation set; *yi* and *ŷ* are the true and predicted prices at the i-th time step, respectively; and $\bar{y}$ is the overall mean of the true prices in the validation set. Lower MAE and RMSE values indicate smaller forecasting errors and thus higher predictive accuracy, whereas an R^2^ value closer to 1 implies a stronger ability of the model to characterize and explain complex temporal dynamics.

### 3.3. Parameter Settings

In this study, the key hyperparameters were determined through a combination of time-series cross-validation and manual tuning to ensure the stability and generalization capability of the model in carbon price forecasting. The specific search space and baseline settings are summarized in Table 2. The model adopts a hierarchical architecture that deeply integrates a bidirectional temporal convolutional network with TimeXer for time-series modeling. In the BiTCN module, one-dimensional convolutions with dilation rates of 1 and 2 are configured to capture multi-scale local contextual features in the carbon price series. The TimeXer module introduces a learnable global token and employs both self-attention and cross-attention mechanisms to strengthen the temporal interactions among multi-source endogenous and exogenous features.

To mitigate internal covariate shift, layer normalization is applied after both the feature extraction and attention layers, while dynamic dropout and weight decay are incorporated to suppress overfitting. To further enhance robustness against extreme price fluctuations, a multi-level dynamic training strategy is adopted during training. Specifically, the Huber loss, which is less sensitive to outliers, is selected as the objective function, and the adaptive moment estimation (Adam) optimizer with gradient clipping is employed to accelerate convergence while maintaining stable parameter updates. For learning rate scheduling, an adaptive decay strategy based on forward-chaining fold-loss stagnation (ReduceLROnPlateau) is adopted, whereby the learning rate is multiplied by a factor of 0.5 when the fold loss fails to improve for three consecutive epochs. Meanwhile, an early stopping mechanism is established with the fold loss as the monitoring criterion and a patience of 8 epochs, with the best model weights automatically restored once early stopping is triggered. This parameter configuration was finalized through iterative time-series cross-validation and manual tuning, thereby preserving the model’s nonlinear fitting capacity while minimizing the risks of overfitting and future information leakage.

## 4. Experimental Results and Discussion

### 4.1. Comparison with Baseline Models

All models are evaluated under the same one-step-ahead forecasting task, input window length, preprocessing procedure, chronological data partition, and evaluation metrics. The reported MAE, RMSE, and R^2^ values are computed after inverse transformation to the original price scale. This design ensures that the comparison focuses on differences in model structure rather than differences in data leakage, scaling, or evaluation protocol.

**Figure 5:**
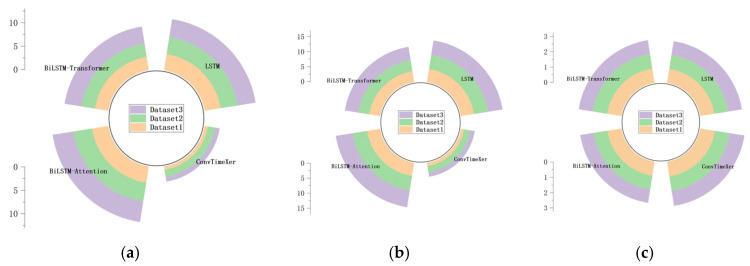
(**a**) Mean absolute error (MAE) evaluation results, (**b**) root mean square error (RMSE) evaluation results, and (**c**) coefficient of determination (R^2^) evaluation results.

To validate the forecasting performance of the proposed model, this study selected the long short-term memory network (LSTM), the bidirectional long short-term memory network combined with Transformer (BiLSTM-Transformer), and the bidirectional long short-term memory network combined with an attention mechanism (BiLSTM-Attention) as baseline models for comparative analysis against ConvTimeXer. These baselines cover recurrent sequence modeling, recurrent-Transformer hybrid modeling, and attention-enhanced recurrent modeling. Considering the non-stationary characteristics of high-frequency carbon-market data, the experiments were conducted using three-fold time-series cross-validation to ensure the generalization ability and reliability of the evaluation results. The detailed numerical results are presented in Table 3, Table 4 and Table 5, and the corresponding comparisons are visually summarized in Figure 5a–c.

Overall, the results reported in Table 3, Table 4 and Table 5 and visually summarized in Figure 5a–c show that ConvTimeXer consistently outperforms the baseline models across the three datasets in terms of MAE, RMSE, and R^2^, demonstrating clear advantages in both forecasting accuracy and robustness. As shown in Figure 5a,b, ConvTimeXer achieves the lowest error values on all datasets, while Figure 5c further indicates that it attains the highest R^2^ values throughout, confirming its superior ability to capture the underlying dynamics of carbon price movements.

Quantitative comparisons in Table 3, Table 4 and Table 5 further reveal the limitations of conventional sequential models when handling the complex fluctuations of carbon prices. LSTM, as a basic recurrent benchmark, yields average RMSE and R^2^ values of 4.6947 and 0.9194, respectively, indicating limited capability in modeling strongly non-stationary series and a tendency to lag around abrupt turning points. BiLSTM-Attention does not show further improvement; instead, it records an average RMSE of 5.0004 and an average R^2^ of 0.9084, suggesting that attention-based local weighting alone remains insufficient to suppress high-frequency noise and maintain stable long-term trend modeling. These patterns are also reflected in Figure 5, where both models remain noticeably behind ConvTimeXer across all three metrics.

By comparison, BiLSTM-Transformer exhibits relatively stronger global feature extraction ability. As reported in Table 3, Table 4 and Table 5, its average MAE decreases to 3.1484 and its average R^2^ increases to 0.9422, outperforming both LSTM and BiLSTM-Attention overall. This tendency is also visible in Figure 5, especially in the R^2^ comparison, where BiLSTM-Transformer consistently ranks second among the four models. Nevertheless, its error values in Figure 5a,b remain substantially higher than those of ConvTimeXer, indicating that its sensitivity to highly localized short-term fluctuations is still insufficient under complex asymmetric market shocks.

In contrast, ConvTimeXer achieves the best overall performance through multi-module collaboration. Across the three datasets, its MAE values are 0.7028, 1.5182, and 1.0444; its RMSE values are 1.0801, 2.1495, and 1.4886; and its R^2^ values are 0.9790, 0.9626, and 0.9843, respectively, as shown in Table 3, Table 4 and Table 5. Correspondingly, Figure 5a–c presents the same conclusion from a visual perspective: ConvTimeXer consistently occupies the most favorable position in all three evaluation dimensions. On average, its MAE, RMSE, and R^2^ reach 1.0885, 1.5727, and 0.9753, respectively. In light of the model architecture, these improvements can be mainly attributed to the following two design aspects.

First, the BiTCN module plays a critical role in capturing short-term local market shocks. For fluctuation sequences whose macro-level trends have been removed by the RevIN module, the model no longer relies on conventional recurrent neural network (RNN) or LSTM recursive structures, but instead adopts a BiTCN architecture combined with dilated convolutions. This module scans the input sequence from both forward and backward directions, thereby alleviating the response lag caused by unidirectional information propagation. Meanwhile, relatively small dilation rates and a kernel size of 3 are more suitable for modeling local fluctuations within short time windows, which facilitates the identification of short-term non-stationary variations. The substantial reductions in MAE and RMSE shown in Table 3, Table 4 and Table 5, as well as the clear error gap in Figure 5a,b, together suggest that BiTCN contributes strongly to filtering local high-frequency noise and extracting short-term fluctuation patterns.

Second, the cross-attention mechanism based on the global token also plays a key role in integrating multidimensional market factors for long-term trend modeling. To avoid the semantic distortion and weight instability that may arise from point-wise attention computation, the model segments the endogenous sequence of length 30 into patches of length 6, thereby preserving the local semantics of consecutive trading days while reducing computational complexity. On this basis, a learnable global token is appended to the end of the patch sequence, and global contextual information of the carbon price itself is aggregated through the self-attention layer. Subsequently, this global token serves independently as a query vector and dynamically interacts with the 14-dimensional exogenous feature set in the cross-attention layer. Compared with directly concatenating endogenous and exogenous variables, this design enables the model to selectively absorb macroeconomic or energy-market information relevant to the current global state of carbon prices, thereby improving both the interpretability of long-term trends and robustness to interference. This advantage is also reflected in Figure 5c, where ConvTimeXer consistently achieves the highest R^2^ values across all datasets, indicating a stronger ability to explain the variance of carbon price fluctuations.

The computational cost of ConvTimeXer can be roughly assessed from its implemented model configuration. In the implemented setting, the input length is 30, the patch length is 6, the hidden dimension is 16, the number of attention heads is 2, and only one encoder layer is used. Therefore, the endogenous sequence is compressed into five patch tokens plus one global token before the attention module. Under this configuration, the proposed model contains approximately 6.6K trainable parameters, which indicates that the implemented model is relatively small in scale.

The main computational cost of ConvTimeXer comes from three components: the bidirectional temporal convolution module, the self-attention over patch and global tokens, and the cross-attention between the global token and exogenous-variable embeddings. Since self-attention is performed over only six tokens rather than the full input sequence, its cost is substantially reduced compared with a standard Transformer operating directly on all time steps. The cross-attention module is also lightweight because only the global token is used as the query to attend to the exogenous-variable sequence. Consequently, the additional memory requirement mainly comes from the attention activations and projected exogenous embeddings, which remain limited under the small hidden dimension and short input window used in this study.

### 4.2. Ablation Study 

To verify the contributions of the core modules in ConvTimeXer, ablation experiments were conducted by removing BiTCN and Cross-Attention, respectively, in order to examine the resulting changes in model performance. All experiments were performed under the same dataset partitioning scheme and hyperparameter settings to ensure the fairness of the comparison and the reliability of the results. The specific ablation settings are as follows:

(1) Without BiTCN: the original module was replaced with a simple dimension-alignment layer, thereby removing the model’s ability to extract local bidirectional temporal features;

(2) Without Cross-Attention: the attention-based interaction pathway between exogenous variables and endogenous features was removed, in order to evaluate the effectiveness of the multi-source external information fusion mechanism.

As shown in Table 6, the ablation results indicate that model performance generally declines after the removal of the core modules, demonstrating that these components play an important role in the overall effectiveness of the framework. To make the differences easier to interpret, the following discussion reports both absolute metric changes and their relative changes compared with the full ConvTimeXer model.

After removing BiTCN, the prediction errors of the model generally increase across all three data subsets. This effect is particularly evident in Dataset 2, which exhibits relatively large market fluctuations: the MAE increases from 1.5182 in the full model to 1.9729, corresponding to an increase of approximately 29.96%, while the RMSE rises from 2.1495 to 2.6708, corresponding to an increase of approximately 24.25%. On average, removing BiTCN increases MAE from 1.0885 to 1.3600 and RMSE from 1.5727 to 1.9492. These results suggest that, by virtue of its bidirectional receptive fields, BiTCN is able to effectively capture local short-term fluctuation patterns in carbon price series. In the absence of this module, the model relies more heavily on downstream global attention, and its capacity to fit sharp short-term fluctuations is weakened.

The removal of Cross-Attention has a more heterogeneous effect. In Dataset 1, the MAE of the ablated model without endogenous-exogenous interaction increases from 0.7028 to 0.8204, corresponding to an increase of approximately 16.73%. In Dataset 2, the MAE increases from 1.5182 to 1.5690, corresponding to an increase of approximately 3.35%. In Dataset 3, the MAE after removing this mechanism is 0.8527, which is lower than 1.0444 for the full model; this indicates that, during certain periods, some exogenous variables may introduce noise or undergo concept drift, thereby interfering with attention-based fusion. Nevertheless, the average RMSE and R^2^ still favor the full model, suggesting that cross-attention contributes to overall forecasting stability and variance explanation even when its effect on MAE varies across market periods.

Using the full ConvTimeXer model as the reference, the average percentage changes provide a clearer comparison of the ablation effects. Removing BiTCN increases the average MAE from 1.0885 to 1.3600, corresponding to a 24.9% increase, and increases the average RMSE from 1.5727 to 1.9492, corresponding to a 23.9% increase; meanwhile, the average R^2^ decreases from 0.9753 to 0.9623, representing a 1.3% reduction in explanatory power. Removing Cross-Attention produces a smaller but still informative change: the average MAE slightly decreases by 0.7%, whereas the average RMSE increases by 4.4% and the average R^2^ decreases by 0.4%. These relative changes suggest that BiTCN has a more direct effect on reducing prediction errors, whereas Cross-Attention mainly helps maintain overall stability and R^2^ performance.

Overall, the ablation results suggest that:

(1) The front-end BiTCN is a critical component for extracting local temporal features and plays a key role in handling sharp short-term market fluctuations;

(2) The cross-attention mechanism enhances the model’s ability to capture complex carbon price dynamics by dynamically integrating multi-source external information, although its marginal contribution may vary when exogenous variables contain noise or concept drift;

(3) These two modules exhibit strong complementarity in temporal feature extraction and multi-source information fusion, jointly supporting the overall performance of ConvTimeXer.

## 5. Conclusions

This study proposed ConvTimeXer, a hybrid forecasting framework that integrates bidirectional temporal convolution with a TimeXer-based cross-attention mechanism for carbon market price forecasting. From the perspective of information structure and complex systems, the model was designed to address three objectives: extracting effective local fluctuation information from endogenous carbon-price dynamics, dynamically fusing heterogeneous exogenous signals, and maintaining leakage-free generalization under non-stationary market uncertainty.

Experimental results on three chronological evaluation datasets demonstrate that ConvTimeXer consistently outperforms the compared baseline models in terms of MAE, RMSE, and R^2^. In particular, the proposed framework achieves average MAE, RMSE, and R^2^ values of 1.0885, 1.5727, and 0.9753, respectively, indicating strong forecasting accuracy and robustness under the adopted one-step-ahead forecasting setting. The ablation study further confirms that BiTCN contributes to local fluctuation modeling, while cross-attention improves the structured use of exogenous information and overall forecasting stability.

Overall, the findings suggest that ConvTimeXer is useful for carbon market price forecasting and may also provide a reference for other multivariate forecasting tasks with non-stationary series and external driving variables. The high R^2^ values should be interpreted in light of the one-step-ahead setting, chronological validation, and the strong temporal continuity of carbon-price series rather than as evidence that the model eliminates all uncertainty in market prediction.

This study also has several limitations. First, the empirical analysis is based on China’s carbon market over a specific observation period. Although the forward-chaining evaluation strategy reduces look-ahead bias, the generalizability of the results should be further tested using longer samples, other regional carbon markets, and periods with different policy regimes or extreme market events. Second, the current experiments focus on one-step-ahead forecasting. Multi-step forecasting may introduce error accumulation and should be examined separately. Third, exogenous variables may contain reporting delays, missing values, frequency mismatches, noise, or concept drift, which may weaken their contribution in some market periods. Finally, although the attention mechanism provides a structured way to combine endogenous and exogenous information, attention weights should not be interpreted as causal evidence. Future research will extend ConvTimeXer to multi-market and multi-horizon settings and explore uncertainty-aware prediction, online updating, and more interpretable exogenous-variable selection. Recent research has shown that a phased-enhancement marine predators algorithm can effectively address global optimization and feature-selection tasks across benchmark datasets and a real-world fraud-detection application [51]. Such metaheuristic approaches may provide a potential direction for optimizing exogenous-variable subsets in future extensions.

## Figures and Tables

**Table 1:** Exogenous variables.

No.	Variable Name
1	EUR/CNY closing exchange rate
2	HKD/CNY closing exchange rate
3	JPY/CNY closing exchange rate
4	USD/CNY closing exchange rate
5	GBP/CNY closing exchange rate
6	Cumulative coal consumption for heating (10,000 tons)
7	Monthly power generation (100 million kWh)
8	China crude oil composite CIF import price index (points)
9	China LNG ex-factory price index (points)
10	CSI 300 closing index
11	CSI 1000 closing index
12	Thermal coal price (4500 kcal/kg) (CNY/ton)
13	Thermal coal price (5000 kcal/kg) (CNY/ton)
14	Thermal coal price (5500 kcal/kg) (CNY/ton)

Note: In Table 1, EUR denotes euro, HKD denotes Hong Kong dollar, JPY denotes Japanese yen, USD denotes United States dollar, GBP denotes pound sterling, CNY denotes Chinese yuan, CIF denotes cost, insurance, and freight, LNG denotes liquefied natural gas, CSI denotes China Securities Index, kWh denotes kilowatt-hour, and kcal denotes kilocalorie.

**Table 2:** Core model hyperparameter settings and search space.

Parameter Category	Parameter Name	Setting/Search Space
Network Architecture	Input look-back window size	30
	Patch length	6
	Transformer hidden dimension	{8, 16, 32}
	Number of self-attention heads	{2, 4}
	Feed-forward network dimension	{16, 32, 64}
	Number of Transformer layers	{1, 2}
Regularization Configuration	Dropout rate	[0.1, 0.4]
	Adam weight decay	1 × 10^−4^
	Gradient clipping threshold	1.0
Training Strategy	Initial learning rate	3 × 10^−4^
	Batch size	16
	Maximum number of training epochs	80
	Number of cross-validation folds	3

**Table 3:** Evaluation results on Dataset 1.

Model	MAE	RMSE	R^2^
LSTM	3.4589	4.4295	0.9261
BiLSTM-Transformer	2.8682	3.6817	0.9445
BiLSTM-Attention	3.5085	4.4309	0.9294
ConvTimeXer	0.7028	1.0801	0.9790

**Table 4:** Evaluation results on Dataset 2.

Model	MAE	RMSE	R^2^
LSTM	3.6574	4.7173	0.9175
BiLSTM-Transformer	2.8952	3.6882	0.9539
BiLSTM-Attention	4.0436	4.9044	0.9129
ConvTimeXer	1.5182	2.1495	0.9626

**Table 5:** Evaluation results on Dataset 3.

Model	MAE	RMSE	R^2^
LSTM	3.9184	4.9373	0.9145
BiLSTM-Transformer	3.6819	4.5498	0.9281
BiLSTM-Attention	4.4730	5.6659	0.8829
ConvTimeXer	1.0444	1.4886	0.9843

**Table 6:** Ablation study results.

Dataset	Model	MAE	RMSE	R^2^
Dataset 1	No BiTCN	0.7571	1.2976	0.9697
	No Cross-Attention	0.8204	1.3362	0.9679
	Full Model	0.7028	1.0801	0.9790
Dataset 2	No BiTCN	1.9729	2.6708	0.9423
	No Cross-Attention	1.5690	2.3049	0.9570
	Full Model	1.5182	2.1495	0.9626
Dataset 3	No BiTCN	1.3499	1.8792	0.9750
	No Cross-Attention	0.8527	1.2850	0.9880
	Full Model	1.0444	1.4886	0.9843
Average	No BiTCN	1.3600	1.9492	0.9623
	No Cross-Attention	1.0807	1.6420	0.9710
	Full Model	1.0885	1.5727	0.9753

## Data Availability

The data that support the findings of this study are available from the third party https://www.cctd.com.cn/, accessed on 26 February 2026. However, restrictions apply to the availability of these data, which were used under license for the current study and are therefore not publicly available.

## Abbreviations

The following abbreviations are used in this manuscript:

BiLSTM	Bidirectional long short-term memory
BiTCN	Bidirectional temporal convolutional network
CEEMD	Complementary ensemble empirical mode decomposition
CNN	Convolutional neural network
FFN	Feed-forward network
LSTM	Long short-term memory
MAE	Mean absolute error
R^2^	Coefficient of determination
RevIN	Reversible instance normalization
RMSE	Root mean square error
RNN	Recurrent neural network
TCN	Temporal convolutional network
VMD	Variational mode decomposition
CSI	China Securities Index
LNG	Liquefied natural gas
